# Childhood intussusceptions at a tertiary care hospital in northwestern Tanzania: a diagnostic and therapeutic challenge in resource-limited setting

**DOI:** 10.1186/1824-7288-40-28

**Published:** 2014-03-11

**Authors:** Phillipo L Chalya, Neema M Kayange, Alphonce B Chandika

**Affiliations:** 1 Department of Surgery, Catholic University of Health and Allied Sciences-Bugando, Mwanza, Tanzania; 2 Department of Paediatrics, Catholic University of Health and Allied Sciences-Bugando, Mwanza, Tanzania

**Keywords:** Intussusception, Children, Pattern, Clinical presentations, Management, Outcome, Tanzania

## Abstract

**Background:**

Intussusception remains a common cause of bowel obstruction in children and results in significant morbidity and mortality if not promptly treated. There is a paucity of prospective studies regarding childhood intussusception in Tanzania and particularly the study area. This study describes the pattern, clinical presentations and management outcomes of childhood intussusception in our setting and highlights the challenging problems in the management of this disease.

**Methods:**

This was a prospective descriptive study of patients aged < 10 years operated for intussusception at Bugando Medical Centre. Ethical approval to conduct the study was obtained from relevant authorities. Data was analyzed using SPSS version 17.0.

**Results:**

A total of 56 patients were studied. The male to female ratio was 3.3: 1. The median age was 6 months. Three-quarter of patients were < 1 year. Etiology was mainly idiopathic in 91.1% of cases. The classic triad of bloody stool, vomiting and abdominal distention/abdominal pain was found in 24 (42.5%) patients. The diagnosis of intussusception was mainly clinically in 71.4% of cases. All patients were treated surgically. Ileo-colic was the most frequent type of intussusception (67.9%). Twenty-six (46.4%) patients required bowel resection. The rate of bowel resection was significantly associated with late presentation > 24 hour (*p* = 0.001). Complication rate was 32.1% and surgical site infection (37.5%) was the most frequent complication. The median length of hospital stay was 7 days. Patients who had bowel resection and those who developed complications stayed longer in the hospital and this was statistically significant (p < 0.001). Mortality rate was 14.3%. Age < 1 year, delayed presentation, associated peritonitis, bowel resection and surgical site infection were the main predictors of mortality (p < 0.001). The follow up of patients was generally poor

**Conclusion:**

Intussusception in our setting is characterized by late presentation, lack of specialized facilities and trained personnel for nonsurgical reduction. Therefore, surgery remains the main stay of treatment in our centre. A high index of suspicion and proper evaluation of patients is essential for an early diagnosis and timely definitive treatment, in order to decrease the morbidity and mortality associated with this disease.

## Background

Intussusception is defined as the invagination of a proximal segment of the intestine into a distal segment of the intestine [1]. It remains a common cause of bowel obstruction in infancy and young children and results in significant morbidity and mortality if not promptly treated [1,2]. The peak age of presentation is 4 to 8 months [2]. In the United States, approximately two-thirds of cases occur below the age of 1 year [3]. Its estimated incidence is approximately 1 case per 2000 live births.

Overall, the male-to-female ratio is approximately 3:1. With advancing age, gender difference becomes marked; in patients older than 4 years, the male-to-female ratio is 8:1 [2,3].

Intussusception is reported to be idiopathic in 90% of cases and is rarely associated with pathological lead points such as Meckel’s diverticulum, solid bowel lesions and intestinal lymphoma [4-6]. Intussusception has also been reported to occur postoperatively and after blunt abdominal trauma [7].

Patients with intussusception often present with a wide range of nonspecific symptoms, including emesis, pain, irritability, and decreased appetite. The classic symptoms of emesis, pain, and bloody stools with or without a mass have been shown to be present in less than a quarter of children, making intussusception a difficult clinical diagnosis [8-10].

The diagnosis of intussusception is challenging due to a wide variety of clinical presentations and overlaps with other abdominal conditions, a situation that often leads to delay in diagnosis [9-11]. It is assumed that the delayed diagnosis of intussusception increases the incidence of surgical treatment and the risk of complications [11-13].

Successful management of intussusception depends on early recognition and diagnosis, adequate fluid resuscitation and prompt reduction [14]. The treatment of intussusception has evolved from primarily operative management to the preference for non-operative reduction with either air or barium contrast. Non-operative reductions of intussusception had been shown to decrease length of hospitalization, shorten recovery, and reduce the risk of complications associated with major abdominal surgery [15]. Reports from some developing countries however indicate that for some ill-defined reasons, operative treatment is still routinely performed for intussusceptions [16-18].

Outcome of treatment in these setting is also reported to be poor compared to the results of treatment to more developed countries [19]. This has been attributed to delayed presentation of disease coupled with lack of trained personnel and unavailability of diagnostic and therapeutic facilities, a common feature in resource-limited setting [16]. Late presentation as a cause of unacceptably high mortality and morbidity rates in intussusception has been documented from many African centers [16,20]. Most of these studies on this condition from our sub-region were retrospective and we therefore embarked on this prospective study to describe our experience with this condition outlining the pattern, clinical presentations and management outcomes of childhood intussusception in a tertiary care hospital setting in northwestern Tanzania and highlight the challenging problem in the management of this disease in our local setting.

## Methods

### Study design and setting

This was a prospective descriptive study of patients aged < 10 years operated for intussusception at Bugando Medical Centre from August 2010 to July 2012. Bugando Medical Centre is a 1000 bed referral hospital located in Mwanza city in northwestern Tanzania on the southern border of Lake Victoria. It is also a teaching hospital for the Catholic University of Health and Allied Sciences (CUHAS). The hospital serves a population of approximately 13 million people from neighboring regions in northwestern Tanzania. More than 50% of this population seeks service from this hospital.

### Study population

The study population included all patients aged 10 years and below who were admitted in the paediatric surgical ward and underwent operation for intussusception at Bugando Medical Centre. Children above 10 years with surgical problems are admitted in the adult surgical wards and therefore were excluded from the study. Recruitment of patients was done at the Accident and Emergency department, in the paediatric medical and surgical wards after initial resuscitation done by the admitting surgical team. Patients who met the inclusion criteria were consecutively enrolled in the study after an informed written consent sought from the parents or guardians.

The preoperative diagnosis of intussusceptions was made clinically and radiologically and the final diagnosis was confirmed at surgery. Only patients who were confirmed at surgery as intussusception were included in the study.

Preoperatively, all the patients recruited into the study had intravenous fluids to correct fluid and electrolyte deficits; nasogastric suction; urethral catheterization and broad-spectrum antibiotic coverage. Adequate hydration was indicated by an hourly urine output of 30 ml/hour. Relevant preoperative investigations included packed cell volume, serum electrolytes, urea and creatinine, blood grouping and cross-matching. Radiological investigations including X-ray abdomen erect and supine, X-ray chest PA-view were done in all patients. Abdominal ultrasound was also performed in some patients as it was not always readily available. Barium enema either for diagnostic or therapeutic purposes and abdominal CT scan were not performed in any of our patients due lack of these facilities and trained personnel in our centre.

After resuscitation, all patients, under general anesthesia were subjected to exploratory laparotomy through either a transverse or midline incision. The operations were performed either by a consultant surgeon or a senior resident under the direct supervision of a consultant surgeon.

Postoperatively patients were kept nil orally till return of bowel sounds and at that time nasogastric tubes were removed. Stitches were removed on 7-10th postoperative day. Patients were followed up until discharge or death. Patients who survived were followed up for up to six months after discharge.

### Data collection

Data were collected using a pre-tested coded questionnaire. Data administered in the questionnaire included; socio-demographic data (age, sex, area of residence), clinical presentation, duration of illness, month of presentation (seasonality), diagnostic modality, treatment modality, operative findings, type of surgical procedure performed and outcome measure (e.g. postoperative complications, death and length of hospital stay).

### Statistical data analysis

The statistical analysis was performed using statistical package for social sciences (SPSS) version 17.0 for Windows (SPSS, Chicago IL, U.S.A). The median (+ IQR) and ranges were calculated for continuous variables whereas proportions and frequency tables were used to summarize categorical variables. Chi-square (χ2) test were used to test for the significance of association between the independent (predictor) and dependent (outcome) variables in the categorical variables. The level of significance was considered as P < 0.05. Multivariate logistic regression analysis was used to determine predictor variables that predict the postoperative complications, hospital stay and mortality

### Ethical considerations

A written informed consent was sought and obtained from parents or relatives and permission to carry out the study was approved by the CUHAS/BMC joint institutional ethic review committee before the commencement of the study.

## Results

### Socio-demographic data

**Figure 1:**
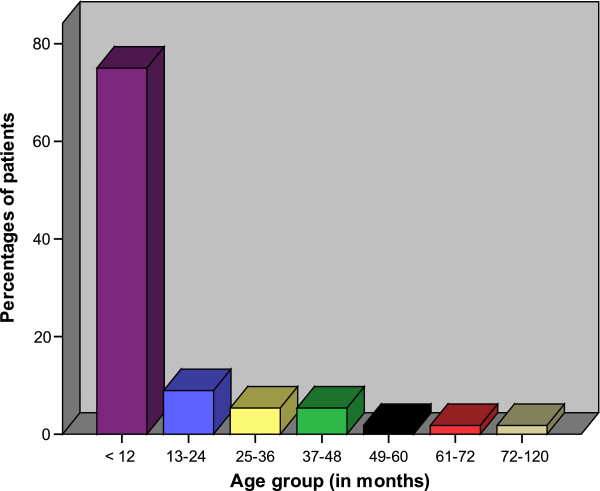
Distribution of patients according to age group.

During the study period, a total of 59 patients were admitted to our centre and underwent laparotomy for acute intestinal obstruction due to intussusceptions. Out of these, 3 patients were excluded from the study due to failure to meet the inclusion criteria. Thus, a total of 56 patients were enrolled in the study. Forty-three (76.8%) were males and 13 (23.2%) females, giving a male to female ratio of 3.3: 1. The age of patients at presentation ranged from 3 months to 120 months with a median age of 6 months (IQR of 4 to 8 months). The peak age incidence was in the age group of 4–8 months. Seventy-five per cent of patients were aged below 1 year (12 months) at the time of presentation (Figure 1). The majority of patients, 45(80.4%) came from the rural areas located a considerable distance from the study area.

### Admission patterns

Of the 56 patients, forty (71.4%) were referred from peripheral hospitals due to an uncertainty in diagnosis or with an established diagnosis for further treatment and the remaining 16 (28.6%) patients were admitted directly at Bugando Medical Centre. Out of 16 patients who were admitted directly to Bugando Medical Centre, 9 (56.3%) were treated for other medical illnesses namely; gastro-enteritis in 4 patients, dysentery and nonspecific abdominal pain in 3 and 2 patients respectively. The decision for referral to surgeons for those who were treated for other illnesses was prompted by setting in of abdominal distention.

### Etiology and seasonality

In this study, the etiology of intussusceptions was idiopathic in 51 (91.1%) patients and the remaining 5 (8.9%) patients showed pathological lead points. The high incidence of intussusceptions was observed during dry months (May to October) and low incidence was observed during rainy seasons (November to April). We could not observe associated high incidence of viral illness such as upper respiratory diseases.

### Clinical presentation

The duration of symptoms ranged from 1–14 days with the median duration of 4 days (IQR of 2 to 8 days). The majority of patients (53.6%) presented between 3 and 5 days (Table 1). Abdominal distention was the most common presenting symptom occurring in 85.7% of cases. Presenting abdominal distension was more common in children with symptoms of > 24 hours duration (80.4% vs 5.4%, p = 0.00). The majority of patients (89.3%) were assessed as dehydrated requiring fluid resuscitation before operation (Table 1). The classic triad of bloody stool/rectal bleeding, vomiting and abdominal distention/abdominal pain was found in 24 (42.5%) patients. Abdominal mass was often difficulty to palpate due to gross or tense abdomen and was only palpated in six (10.4%) patients.

**Table 1:** Distribution of patient according to clinical presentation

**Clinical presentation**	**Frequency**	**Percentages**
***Duration of symptoms***		
< 24 hours	7	12.5
1-2 days	14	25.0
3-5 days	30	53.6
>5 days	5	8.9
***Symptoms***		
Abdominal distention	48	85.7
Vomiting	42	75.0
Bloody stool	18	32.1
Diarrhea	13	23.2
Constipation	9	16.1
Fever	8	14.3
Abdominal pain	8	14.3
Bleeding per rectum	6	10.7
Prolapsing rectal mass	2	3.6
***Signs***		
Dehydration	50	89.3
Peritonism	14	25.0
Abdominal mass	6	10.7
Rectal mass	2	3.6

### Diagnosis of intussusception

The preoperative diagnosis of intussusception was made clinically in 40 (71.4%) patients, radiologically (only by abdominal ultrasound in 5 patients) in 5 (10.7%) and surgically at laparotomy for intestinal obstruction in 11 (19.6%) patients. The specific diagnosis of intussusceptions was then confirmed at surgery. Contrast enema was not carried out in any of our patients due to lack of this facilities and trained personnel at our centre. Plain abdominal x-rays performed in all patients (100%) revealed features suggestive of intestinal obstruction in 51 (91.1%) patients. None of the plain abdominal x-rays detected intussusception.

### Treatment modality

All patients (100%) in this study were treated surgically after resuscitation. Ileo-colic was the most frequent type of intussusception accounting for 67.9% of patients (Table 2). Gangrenous bowel and perforations were reported in 14 (25.0%) and 5(8.9%) patients, respectively. Five (8.9%) had pathological lead points such as lymphoid hyperplasia of Peyer’s patches in 3 patients and intestinal polyps and post-appendiceal stump in 1 patient each, respectively. Successful manual reduction was the most common type of surgical procedure performed in 53.6% of cases. Twenty-six of 56 (46.4%) children required bowel resection with either primary anastomosis (42.8%) or stoma creation (3.6%) (Table 2). In the two patients who had bowel resection and stoma creation, one had ileostomy due to resection of gangrenous terminal ileum and the other patient had colostomy due to resection of the gangrenous colonic intussusceptum in patients who had colic-colic intussusception. Of the 26 patients who required bowel resection, 14(53.8%) had gangrenous bowel, 8 (30.8%) had failed manual reduction and 5 (19.2%) had perforations. The rate of bowel resection was significantly associated with late presentation > 24 hour (p = 0.001). Non-operative treatment was not attempted in this series.

**Table 2:** Distribution of patients according to type of intussusception & type of surgical procedure performed (N = 56)

**Variables**	**Number of patients**	**Percentages**
***Type of intussusception***		
Ileo-colic	38	67.9
Colic-colic	14	25.0
Ileo-ileal	4	7.1
***Type of surgical procedure performed***		
Successful manual reduction	30	53.6
Resection and primary anastomosis	24	42.8
Resection and stoma creation	2	3.6

### Treatment outcome

A total of 24 (42.9%) postoperative complications were recorded in 18 (32.1%) patients. Of these, surgical site infection was the most frequent postoperative complications accounting for 37.5% of cases (Table 3). Out of these postoperative complications, abdominal abscess, wound dehiscence and recurrent intussusceptions required surgical treatment and the rest were successfully treated conservatively.

**Table 3:** Distribution of patients according to postoperative complications (N = 24)

**Postoperative complications**	**Frequency**	**Percentages**
Surgical site infection	9	37.5
Pneumonia	4	16.7
Peritonitis/abdominal abscess	3	12.5
Wound dehiscence	3	12.5
Anastomitic breakdown	3	12.5
Urinary tract infection	2	8.3
Septicemia	2	8.3
Recurrent intussusceptions	2	8.3
Paralytic ileus	1	4.2

The overall length of hospital stay ranged from 1 day to 24 days with a median of 14 days (IQR of 12 to 16 days). The length of hospital stay for non-survivors ranged from 1 day to 8 days with a median of 3 days (IQR of 1 to 5 days). Median length of hospital stay was significantly longer in patients who had bowel resection (16 days vs 9 days); *p* = 0.012) and those who developed postoperative complications (14 days vs 10 days; *p* = 0.003).

Eight patients died giving a mortality rate of 14.3%. According to multivariate logistic regression analysis, age < 1 year (OR = 1.5, 95% C.I. (1.1-8.5), p = 0.021), delayed presentation (> 24 hours) (OR = 0.3, 95% CI (0.1- 0.9), p = 0.011), associated peritonitis (OR = 5.5, 95% CI (3.4- 8.8), p = 0.004), bowel resection (OR =7.4, 95% CI (5.9-10.7), p = 0.001), surgical site infection (OR = 1.5, 95% CI (1.2-4.7), p = 0.022) were the main predictors of mortality.

### Follow up of patients

**Figure 2:**
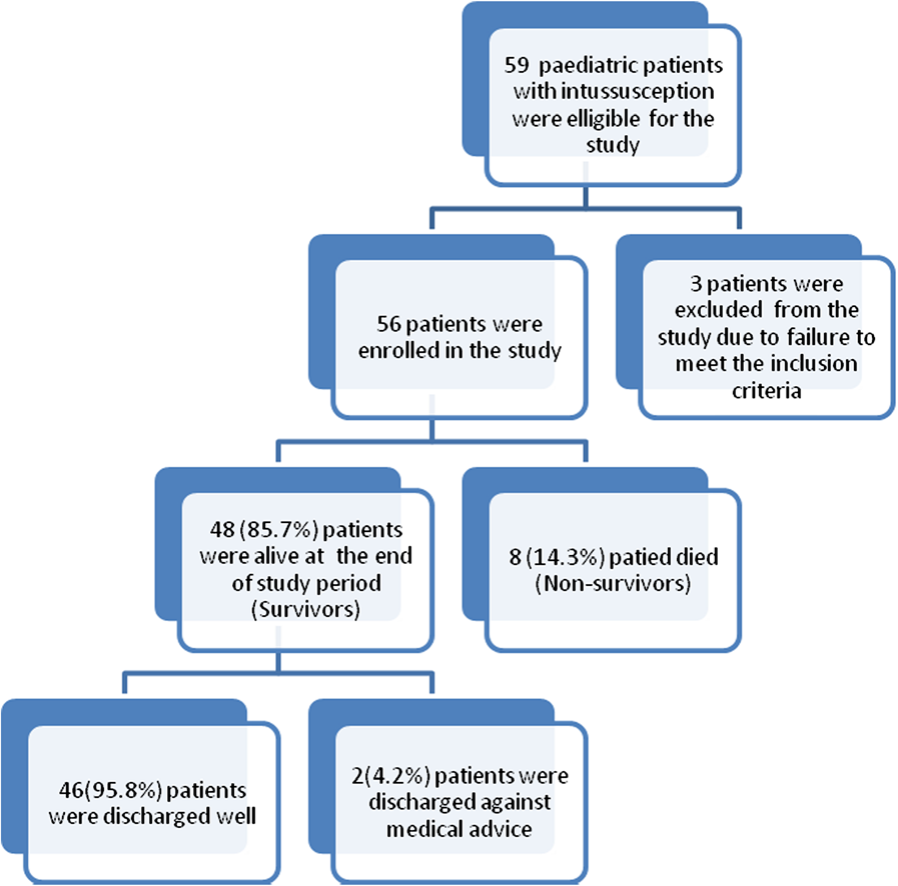
Flow chart showing the clinical outcome of paediatric patients presenting with intussusception at Bugando Medical Centre.

Of the 48 survivors, forty-six (95.8%) patients were discharged well and the remaining two (4.2%) patients were discharged against medical advice. No patient among survivors in this study had permanent disabilities (Figure 2). Of the survivors, twenty-three (47.9%) patients were available for follow up at three to six months after discharge and the remaining 25 (52.1%) patients were lost to follow up.

## Discussion

Intussusception is a common childhood problem that results in serious morbidity and mortality throughout the world and is one of the more common causes of intestinal obstruction in infancy and young children [1]. In this review, three-quarters of patients were in the first year of life which is in agreement with other studies done elsewhere [1,16,21-24], but at variant with other reports in Nigeria that associated childhood intussusception with the above 5 years age group [25,26]. Also, Elebute and Adesola [27] reported a high incidence of intussusception in children older than one year of age. This study showed that males were more affected than females with a male to female ratio of 3.3:1 which is comparable to the results of other workers [1,2,16,17,20,28-30]. Other authors reported female predominance [31]. However the exact reason for this age group and gender differences is not known.

In this study, more than eighty percent of the patients came from the rural areas located a considerable distance from the study area which is in keeping with other studies [16,17,30], This observation has an implication on accessibility to health care facilities and awareness of the disease.

The etiology of intussusception in children remains a dilemma as it largely idiopathic in more than 90% of the cases [3,5-7,16,30,31]. In our study, idiopathic intussusceptions was reported in 91.1% of patients and the remaining 8.9% of cases showed pathological lead points such as lymphoid hyperplasia of Peyer’s patches in 3 patients and intestinal polyps and post-appendicectomy in 1 patient each, respectively. The incidence of idiopathic intussusception has been reported in several studies to have a seasonal variation, with peaks coinciding with the peak incidence of viral respiratory tract infections and diarrheal diseases [32,33]. In the present study, we observed a significant seasonal variation with a definite increase during the dry months (May to October), and a low incidence during rainy season (November to April). Other authors also reported similar seasonal variation [16,22,26,30]. Viral illness such as gastroenteritis, upper respiratory infections and other flu-like illness are known predisposing factors to idiopathic intussusception [34,35]. An increase in the incidence of these diseases during dry months may be responsible for seasonal variation observed. However, an increase in the incidence of these associated diseases was not documented in our patients.

The clinical presentation of intussusception in our patients is not different from those in other studies performed in developing countries [16,17,20,27,30,31,36]. Most of the patients presented later than four days to the surgeon, a figure which is longer compared to reports from developed countries [4,9,15,19] that give duration in terms of hours. Some of our patients presented early but were treated for other medical illnesses in the pediatric wards and were referred to surgeons when abdominal distention set in indicating lack of awareness of the condition among health providers in our setting and in other similar studies in resource limited setting [16,30,36]. Most of the patients in this study were therefore picked in the late stages of disease progression when absolute intestinal had set in. The reasons for late presentation in the present study may be attributed to the fact that the diagnosis of intussusception in its initial stages is usually difficult due to vague and non-specific symptoms as a result patients remain undiagnosed for prolong periods, receiving symptomatic treatment in the pediatric wards or in the peripheral hospitals and subsequently present to surgeons late when intestinal obstruction had set in. The unequal distribution of expertise due to low doctor patient ratio in resource-limited setting renders the diagnosis of intussusceptions at the health centers and most peripheral hospitals difficult to achieve as primary health care workers in these areas may not adequately handle challenges when faced with relatively commoner differentials e.g. gastroenteritis in a daily basis. This calls for an urgent awareness campaign among doctors, nurses, and parents in our environment to raise the index of suspicion and increase the rate of early presentation in this condition. Only 42.5% of the patients reported with the classical triad of vomiting, colicky abdominal pain and red currant jelly stools. The low reporting of classical presentation has been shown by other studies from Africa [16,20]. Kuremu [30] found such symptoms in 17% of his patients. Other authors reported 33%, 32%and 7.5% [17,37,38] among their cases. Primary health care providers have to be aware to this, as many patients may be missed in the critical time.

The diagnosis of intussusceptions varies substantially by region. Whereas in developed countries, the diagnosis of intussusceptions is made radiologically (air-contrast enema, abdominal ultrasound, computered tomography etc.) in over 95% of cases, the diagnosis of intussusceptions in developing countries is made clinically or at surgery in the majority of cases [16,17,30]. This observation is reflected in our study where more than 70% of our patients were diagnosed clinically. Timely diagnosis in this condition is usually dependent on the primary physicians rather than surgeons. Because of the often nonspecific and diverse presenting signs and symptoms, primary physicians must continue to have a high index of suspicion to diagnose children with intussusception. These nonspecific presenting signs and symptoms of patients have been addressed by some pediatric radiologists through the use of ultrasound to screen for intussusception before invasive techniques [39,40]. However, Ultrasonography was not always readily available in this series and was employed in only 10.7% of patients as it would not have significantly influenced the course of surgical therapy.

In keeping with other studies from developing countries [16,17,20,27,30,31,36], surgical intervention was the main stay of treatment performed in all of our patients. This is in contrary to studies in developed countries where intussusception is usually managed by nonsurgical reduction and surgical reduction is indicated only when perforation of bowel is suspected or when radiological reduction fails [41-43]. Nonsurgical reductions of intussusception had been shown to decrease length of hospitalization, shorten recovery, and reduce the risk of complications associated with major abdominal surgery [15]. However, despite the reports on the benefit of nonsurgical treatment, surgery still has a definite role in the management of intussusceptions. Such cases with features of peritonitis at presentation, or those that fail to reduce with non-operative means and patients with pathological lead points and/or bowel complications may invariably require surgery [44]. In this study, nonsurgical reduction was not performed due to late presentation and dearth of specialized facilities and trained personnel. The lack of qualified personnel in the radiological unit coupled with lack of enthusiasm in radiological reduction has shifted much work to the surgeon.

In the present study, ileo-colic intussusception was the most frequent type seen at laparotomy. This is in agreement with other studies performed elsewhere [1,5,16,17,20,30,31,33],[36]. We could not establish the reason for this observation.

The rate of bowel resection in our study was found to be 46.4%, a figure which is higher than 33% and 39% reported in Kenya [30] and Tanzania respectively [16]. The higher rate of bowel resection in our study is attributed to the late presentation to the surgeon, which is a reflection of the low level of health awareness in our community. The late presentation may lead to increasing edema of the bowel wall and advancing intussusception, which clearly reduces the chances of nonsurgical reduction [44]. It is obvious that the duration of symptoms displays a significant factor of morbidity for complications and, necessarily, bowel resection. Intensive health education with a view of promoting increased health awareness and encouraging early presentation of patients to hospital will reduce the bowel resection rate and morbidity and mortality associated with the disease.

The presence of complications has an impact on the final outcome of patients presenting with intussusception. In keeping with other studies [1,16,17,20,30,36,44], surgical site infection was the most common postoperative complications in the present study. In the present study, we found a total recurrence rate of 8.3%, which is consistent with the previously published recurrence rates of 8 - 10% [45-47]. In this study, the presence of complications was found to be associated with high mortality and prolonged length of hospital stay.

The overall median duration of hospital stay in the present study was 14 days which is higher than that reported by Ekenze *et al.*[44] in Nigeria. The reasons for prolonged length of hospital stay in our can be explained by the presence of large number of patients with postoperative complications and bowel resection in our study. However, due to the poor socio-economic conditions in most developing countries including Tanzania, the duration of inpatient stay for our patients may be longer than expected.

The overall mortality rate in this study was 14.3%, a figure which is higher than 8.5% reported by Ekenze *et al.*[44] in Nigeria. Harouna *et al.*[48] observed a high mortality rate of 55% among cases of paediatric intussusceptions in Niger. This was attributed to delayed presentation and advanced peritonitis, coupled with inadequate facilities to manage these challenging cases. The high mortality rate in our study was attributed to age < 1 year, delayed presentation (> 24 hours), associated peritonitis, bowel resection and presence of surgical site infection. Addressing these factors responsible for high mortality in our patients is mandatory to be able to reduce mortality associated with this disease.

The follow-up of patients in this study was generally poor as more than fifty percent of patients were lost to follow up, and data on long-term complications were not available. This observation concurs with other studies performed in developing countries [16,20,30]. Poor follow-up of patients in our study may be explained by the fact that the majority of patients were lost to follow-up at the end of the study period.

The high morbidity and mortality rates in this study are attributed to delayed presentation of disease, lack of diagnostic and therapeutic facilities and trained personnel seen in developed world. Findings from this study is a typical example of diagnostic and therapeutic challenges seen in most developing countries where delayed presentation of the disease coupled with lack of diagnostic and therapeutic facilities and trained personnel for non-operative reduction and poor referral system are among the hallmarks of the disease [16,20].

## Conclusion

The management of childhood intussusceptions in our setting is associated with high morbidity and mortality, mostly due to late presentation, poor referral system and lack of diagnostic and therapeutic facilities. Surgery remains the main stay of treatment in our centre due late presentation and dearth of specialized facilities and trained personnel for non-operative reduction. It is hoped that early presentation and availability of specialized facilities and trained personnel for non-operative reduction will encourage the use of this treatment modality on selected cases in our center. A high index of suspicion, improved referral system, proper evaluation of patients is essential for an early diagnosis and timely definitive treatment, in order to decrease the morbidity and mortality associated with this disease. Factors that were found to be associated with high morbidity and mortality in this study need to be addressed.

## Competing interests

The authors declare that they have no competing interests. The study had no external funding. Operational costs were met by authors.

## Authors’ contributions

ABC & PLC participated in study design, literature search, data analysis, manuscript writing and editing. In addition, PLC submitted the manuscript. NMK participated in the initial management of patients, data analysis, manuscript writing & editing. All the authors read and approved the final manuscript.
